# Effects of Glucose with Casein Peptide Supplementation on Post-Exercise Muscle Glycogen Resynthesis in C57BL/6J Mice

**DOI:** 10.3390/nu10060753

**Published:** 2018-06-11

**Authors:** Yutaka Matsunaga, Yasuyuki Sakata, Takumi Yago, Hirohiko Nakamura, Takashi Shimizu, Yasuhiro Takeda

**Affiliations:** Wellness & Nutrition Science Institute, Morinaga Milk Industry Co., Ltd., 1-83-5 Higashihara, Zama-City 252-8583, Kanagawa Prefecture, Japan; ys-sakata@morinagamilk.co.jp (Y.S.); takumi-yago131@morinagamilk.co.jp (T.Y.); hi_nakam@morinagamilk.co.jp (H.N.); t_simizu@morinagamilk.co.jp (T.S.); ya_taked@morinagamilk.co.jp (Y.T.)

**Keywords:** casein peptide, muscle glycogen, post-exercise, insulin signaling, energy expenditure

## Abstract

Numerous studies have reported that post-exercise ingestion of carbohydrates with protein supplementation can enhance glycogen recovery. However, few reports have focused on the degrees of degradation of the ingested proteins due to post-exercise glycogen resynthesis. Accordingly, the aim of this study was to clarify the effects of differences in protein degradation on muscle glycogen recovery. Male seven-week-old C57BL/6J mice performed a single bout of 60-min treadmill running exercise and were then orally administered glucose (Glu; 1.5 mg/g body weight (BW)), glucose with casein peptide (Glu + Pep; 1.5 + 0.5 mg/g BW) or its constituent amino acid mixture (Glu + AA; 1.5 + 0.5 mg/g BW). At 120 min after supplementation, the soleus muscle glycogen content in the Glu and Glu + AA groups was significantly higher than that immediately after exercise; however, no such difference was observed in the Glu + Pep group. Blood substrate concentration and insulin signaling did not differ among the three groups. Furthermore, energy expenditure during the recovery period in the Glu + Pep group was significantly higher than that in the Glu and Glu + AA groups. These findings suggest that post-exercise co-ingestion of glucose and casein peptide might delay glycogen resynthesis, at least in part through increased energy expenditure caused by casein peptide ingestion.

## 1. Introduction

One of the major causes of fatigue following exercise is depletion of glycogen. Glycogen decreases along with the passage of exercise time [1] and carbohydrate availability is dependent on exercise intensity [2,3]. Furthermore, muscle glycogen content is related to exercise performance [4]. Thus, it is important to enhance glycogen storage before exercise, and to promote glycogen resynthesis after exercise.

Post-exercise nutritional supplementation is necessary for optimal muscle glycogen recovery. To enhance post-exercise muscle glycogen resynthesis, carbohydrate ingestion is required [5]. In addition, previous studies demonstrated that carbohydrates with protein supplementation enhanced the rate of muscle glycogen storage after exercise compared with ingestion of carbohydrates alone [6,7]. These results suggest that protein supplementation promotes glycogen recovery by carbohydrate intake. Furthermore, protein hydrolysate affects glycogen storage. For example, Morifuji et al. [8,9] reported that branched-chain amino acid-containing dipeptides stimulated glucose uptake in L6 myotubes and isolated skeletal muscles, and that post-exercise carbohydrates with whey protein hydrolysate supplementation increased skeletal muscle glycogen levels compared with carbohydrates with whey protein and/or branched-chain amino acid ingestion. These reports suggest that whey protein hydrolysate has bioactive functions, and glycogen storage might be affected by the degrees of degradation of the ingested protein. Morifuji et al. [9] also reported that casein hydrolysate did not have a significantly greater glycogen recovery effect as compared with whey protein hydrolysate. However, it is not clear whether these results were due to a difference in the amino acid composition or a unique effect of casein peptide ingestion. Deglaire et al. [10] reported that hydrolyzed casein intake increases the insulin concentration and area under the curve (AUC) compared with intact casein intake. In addition, we previously reported that pre-exercise casein peptide intake improved glucose tolerance in high-fat diet-fed mice [11]. Thus, we hypothesized that casein peptide ingestion might increase glucose uptake from the blood to muscle and enhance glycogen resynthesis to a greater degree than that after ingestion of its constituent amino acid mixture. In addition, to date, only differences in the degree of protein degradation have been investigated with respect to the influence on energy supply and synthesis, whereas energy consumption during the recovery period has not yet been clarified. Thus, the aim of this study was to determine the specific effects of casein peptide on post-exercise muscle glycogen resynthesis compared with those of its constituent amino acids from the aspects of both energy synthesis and consumption.

## 2. Materials and Methods

### 2.1. Ethical Approval

All experimental protocols were approved by the animal experimental committee of the Institutional Animal Care and Use Committee of Morinaga Milk Industry Co., Ltd.

### 2.2. Experimental Animals and Materials

Male seven-week-old C57BL/6J mice were obtained from Clea Japan Inc. (Tokyo, Japan). All mice were housed individually in an environment maintained at 23 °C with a 12-h light–dark cycle (light on from 8:00 until 20:00) and were provided with food (3.291 kcal/kg; 54.7% carbohydrates, 18.8% proteins, 3.9% fat, 6.9% ash, 6.6% fibers, and 9.2% moisture) and water ad libitum. All mice were provided with MR stock, which does not contain milk-derived proteins (Nosan, Kanagawa, Japan). Casein peptide was obtained from Morinaga Milk (Tokyo, Japan) and its constituent amino acids were obtained as follows: lysine (L8662) and asparagine (A0884) from Sigma-Aldrich (St. Louis, MO, USA); threonine (204-01322), valine (224-00084), methionine (137-01605), cysteine (039-20652), isoleucine (125-00865), leucine (128-00855), phenylalanine (169-01303), tyrosine (200-03563), histidine (084-00682), aspartic acid (015-04831), serine (199-00402), glutamic acid (072-00501), glutamine (076-00521), proline (161-04602), glycine (077-00735), alanine (010-01042), and arginine (019-04611) from Wako (Osaka, Japan); and tryptophan (356-07) from Nacalai Tesque (Kyoto, Japan). The general characteristics and amino acid compositions of casein peptide are shown in Table 1. We used a mixture containing equal proportions of glutamic acid and glutamine, and a mixture containing equal proportions of aspartic acid and asparagine for the amino acid mixture treatment group in this study.

### 2.3. Experimental Procedures

**Figure 1:**
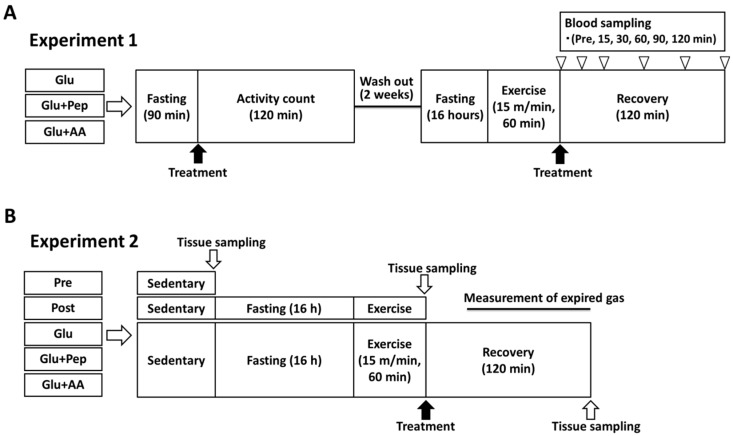
Experimental procedure. (**A**) Experiment 1: Assessments of locomotor activity and blood substrate concentrations after supplementation. (**B**) Experiment 2: Evaluation of muscle glycogen and measurement of expired gas. Pre: pre-supplement and sedentary group. Post: immediately post-exercise group. Glu: glucose ingestion group. Glu + Pep: glucose with casein peptide ingestion group. Glu + AA: glucose with amino acid ingestion group.

Figure 1 provides a schematic overview of the experimental procedures.

#### 2.3.1. Experiment 1

Following an acclimation period of 1 week, mice (*n* = 21) were randomly divided into three treatment groups as follows: glucose ingestion group (Glu, *n* = 7; 1.5 mg/g body weight (BW)), glucose + casein peptide ingestion group (Glu + Pep, *n* = 7; 1.5 + 0.5 mg/g BW), and glucose + amino acid ingestion group (Glu + AA, *n* = 7; 1.5 + 0.5 mg/g BW). The mice orally ingested glucose with or without casein peptide/amino acid mixture after fasting (90 min), and then the activity levels at rest were evaluated during 120 min. After a 2-week washout period, the mice were fasted for 16-h and then were subjected to an endurance exercise (15 m/min, 60 min) to deplete glycogen. Exercise intensity and fasting duration were determined based on the previous study [12]. The mice then received the supplementation treatment and the blood samples were collected from the tail vein (at 0, 15, 30, 60, and 120 min). Blood samples were centrifuged (13,000 rpm, 10 min), and the plasma fractions were rapidly frozen in liquid nitrogen and stored at −80 °C until further analysis.

#### 2.3.2. Experiment 2

Following an acclimation period of 1 week, mice (*n* = 37) were randomly divided into five treatment groups as follows: pre-control group (Pre, *n* = 6), post-exercise group (Post, *n* = 7), glucose ingestion group (Glu; 1.5 mg/g BW, *n* = 8), glucose + casein peptide ingestion group (Glu + Pep; 1.5 + 0.5 mg/g BW, *n* = 8), and glucose + amino acid ingestion group (Glu + AA; 1.5 + 0.5 mg/g BW, *n* = 8). Exercise intensity, fasting duration, and treatment dose were the same as those applied in Experiment 1. During the recovery period (30–120 min), the expiratory gas was collected from the mice, and then the animals were anesthetized using isoflurane and euthanized by post-caval venous blood collection. Tissues were dissected, rapidly frozen in liquid nitrogen, and stored at −80 °C until further analysis.

### 2.4. Analysis

#### 2.4.1. Locomotor Activity

Spontaneous locomotor activity after ingestion was measured using an infrared sensor (LOCOMO, Melquest, Toyama, Japan). We counted the interruptions of latticed infrared beams during movement of a mouse in the cage inside the sensor, which was considered to reflect the spontaneous locomotor activity.

#### 2.4.2. Blood and Plasma Substrate Concentrations

Blood glucose was measured using an auto analyzer (Glutest-Ace; Arkray Inc., Kyoto, Japan). Plasma insulin was measured using an ELISA kit (Ultrasensitive mouse insulin ELISA; Mercodia, Uppsala, Sweden).

#### 2.4.3. Liver and Muscle Glycogen Concentrations

The liver, plantaris muscle, and soleus muscle glycogen levels were measured using the phenol-sulfuric acid method as described previously [13]. The whole soleus muscle was weighed and added to 300 µL of 30% KOH with Na_2_SO_4_ to completely dissolve the tissue. Homogenized solutions were mixed with 360 µL ethanol and placed on ice for 30 min, followed by centrifugation (4 °C, 6000 rpm, 20 min) and the supernatants were removed. The glycogen-containing precipitate was dissolved in distilled water. The solution was reacted with phenol (32079-10, Kanto Chemical, Tokyo, Japan) and sulfuric acid (Kokusan Chemical, 2122723, Tokyo, Japan) for 15 min and absorbance was measured at 490 nm.

#### 2.4.4. Western Blot Analysis

The liver, plantaris muscle, and soleus muscles of the mice were homogenized using a lysis buffer (1% Triton X-100, 50 mM Tris-HCl, 1 mM EDTA, 1 mM EGTA, 50 mM sodium fluoride, 10 mM sodium β-glycerol phosphate, 5 mM sodium pyrophosphate, and 2 mM dithiothreitol; pH 7.5) containing protease inhibitor (1183617001, Complete Mini EDTA-free, Roche Life Science, Indianapolis, IN, USA) and phosphatase inhibitor (04906837001, PhosSTOP phosphatase inhibitor cocktail, Roche Life Science). Protein contents were measured using the Bradford method [14]. We loaded an equal amount (10 μg protein) of homogenized tissue and separated the proteins using standard sodium dodecyl sulfate-polyacrylamide gel electrophoresis procedures (456-1096, 4–20% polyacrylamide gels, Bio-Rad, Hercules, CA, USA), and transferred them onto a polyvinylidene difluoride membrane (1704156, Trans-Blot^®^ Turbo™ Mini PVDF Transfer Packs, Bio-Rad). The membranes were blocked with 3–5% bovine serum albumin in Tris-buffered saline (T5912, Bio-Rad) containing 0.1% Tween 20 (TBST; 170-6531, Bio-Rad) for 1 h, and then incubated overnight at 4 °C with the following primary antibodies: phospho-Akt [p-Akt Ser473, 9271, Cell Signaling Technology (CST), Tokyo, Japan, 1:4000], total-Akt [t-Akt, 9272, CST, 1:4000], phospho-glycogen synthase [p-GS Ser641, 3891, CST, 1:4000], total-glycogen synthase [t-GS, 3893, CST, 1:4000], phospho-AMPKα [p-AMPK Thr172, 2535, CST, 1:4000], and total-AMPKα [t-AMPK, 5832, CST, 1:4000]. After incubation, the membranes were washed in TBST, incubated for 1 h at room temperature with secondary antibodies (A102PT, American Qualex, CA, USA), and washed again in TBST. Chemiluminescent reagents (RPN 2232 and RPN 2109, GE Healthcare Japan, Tokyo, Japan) were used to facilitate blot detection. The blots were scanned and quantified using ChemiDoc XRS (170-8071, Bio-Rad, Hercules, CA, USA) and Quantity One (170-9600, Bio-Rad).

#### 2.4.5. Expired Gas Analysis

The expired gas during the post-exercise recovery period was measured using the O_2_/CO_2_ metabolism measuring system for small animals (MK-5000RQ, Muromachi kikai, Tokyo, Japan). The energy expenditure was calculated using the following formula according to a previous study [15]. Energy expenditure (kcal/min) = (3.91 VO_2_ (L/min) + 1.10 VCO_2_ (L/min)) and normalized to metabolic body size (kg^0.75^).

#### 2.4.6. Statistical Analysis

All data are expressed as mean ± standard error of the mean. All statistical analyses were carried out using JMP 13. In the experiments that involved glucose concentration, insulin concentration and expired gas analysis, two-way analysis of variance (ANOVA) was performed to examine the effects of time and dietary supplements. For the other experiments, statistical analysis was performed using one-way ANOVA. When differences were found to be significant, comparisons were made using Tukey’s post-hoc test. Statistical significance was defined as *p* < 0.05. 

## 3. Results

### 3.1. Experiment 1

#### 3.1.1. Locomotor Activity

**Figure 2:**
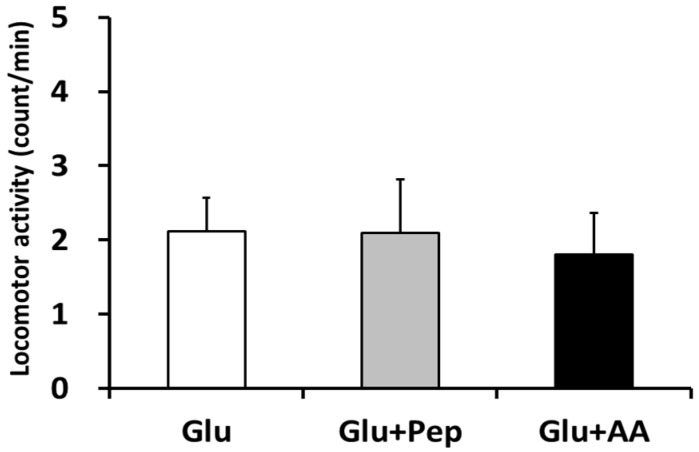
Locomotor activity level during the first 120 min after supplementation. Values are presented as mean ± SEM.

We measured spontaneous locomotor activity to confirm the effects of supplement ingestion on the activity level. Locomotor activity after ingestion was similar among all groups (Figure 2).

#### 3.1.2. Blood Substrate Concentrations

**Figure 3:**
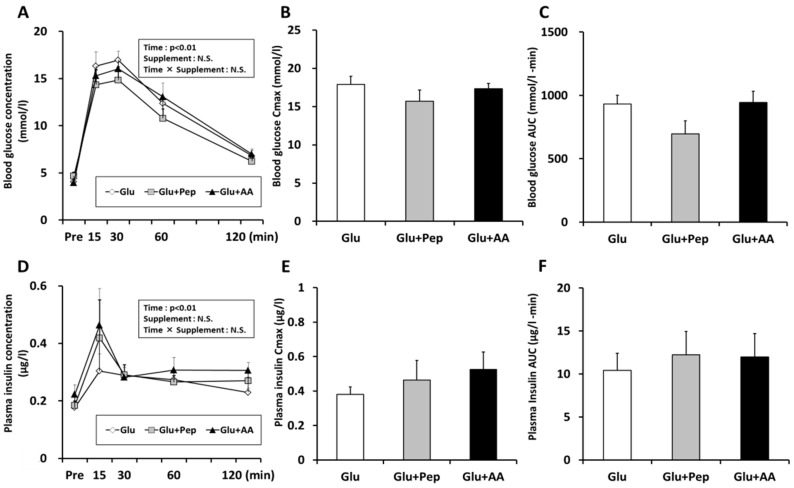
Blood substrate concentration. (**A**) Blood glucose concentration; (**B**) Blood glucose Cmax; (**C**) Blood glucose AUC; (**D**) Plasma insulin concentration; (**E**) Plasma insulin Cmax; (**F**) Plasma insulin AUC. Values are presented as mean ± SEM.

No significant main effects of the supplements or time were observed on variations in blood glucose concentration (Figure 3A). After ingestion, blood glucose levels immediately increased, but the blood glucose Cmax, and AUC were not significantly different among the groups (Figure 3B,C). Similarly, no significant main effects of supplements or time were observed on the plasma insulin concentration (Figure 3D). Although plasma insulin Cmax and AUC in the Glu + Pep (+22% and +38%) and Glu + AA (+38% and 15%) groups were increased compared with those of the Glu group, the differences were not statistically significant (Figure 3E,F).

### 3.2. Experiment 2

#### 3.2.1. Liver and Muscle Glycogen Concentrations

**Figure 4:**
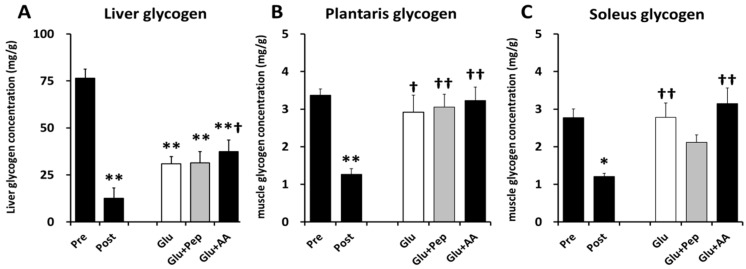
Glycogen content in the liver and muscles. (**A**) Liver glycogen concentration; (**B**) Plantaris muscle glycogen concentration; (**C**) Soleus muscle glycogen concentration. Values are presented as mean ± SEM. * significantly different from the pre-exercise (Pre) group (*p* < 0.05); ** significantly different from the Pre-group (*p* < 0.01); ^†^ significantly different from the post-exercise (Post) group (*p* < 0.05); ^††^ significantly different from the Post group (*p* < 0.01).

The liver glycogen concentration in the Pre (control) group was significantly higher than those of the other groups (Figure 4A, *p* < 0.01). In the recovery period, the liver glycogen concentration in the Glu + AA group was higher than that in the Post group (Figure 4A, *p* < 0.05). In the plantaris muscle, the glycogen concentration in the Post group was significantly lower than that in the Pre-group (Figure 4B, *p* < 0.01). In addition, the glycogen concentrations in the Glu (*p* < 0.05), Glu + Pep (*p* < 0.01), and Glu + AA (*p* < 0.01) groups were higher than those in the Post group (Figure 4B). The soleus muscle glycogen concentration in the Post group was significantly lower than that in the Pre-group (Figure 4C, *p* < 0.05). However, in the recovery period, the muscle glycogen contents in the Glu and Glu + AA groups were higher than that in the Post group (Figure 4C, *p* < 0.01), whereas there was no difference between the Glu + Pep and Post groups (Figure 4C).

#### 3.2.2. Insulin Signaling and Glycogen Synthesis

**Figure 5:**
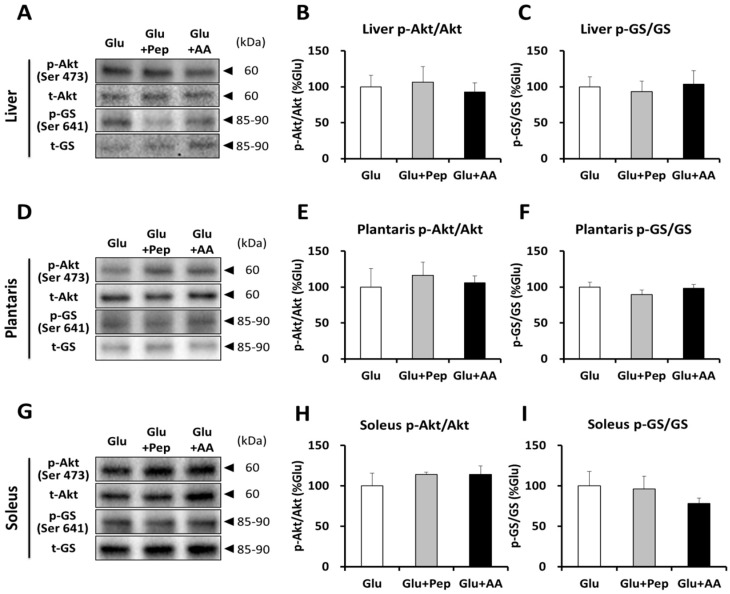
Akt and glycogen synthase phosphorylation state. (**A**) Representative western blots for p-Akt, total-Akt, p-GS, and total GS in the liver; (**B**) Akt phosphorylation state in the liver; (**C**) GS phosphorylation state in the liver; (**D**) Representative blots for p-Akt, total-Akt, p-GS, and total GS in the plantaris muscle; (**E**) Akt phosphorylation state in the plantaris muscle; (**F**) GS phosphorylation state in the plantaris muscle; (**G**) Representative blots for p-Akt, total-Akt, p-GS, and total GS in the soleus muscle; (**H**) Akt phosphorylation state in the soleus muscle; (**I**) GS phosphorylation state in the soleus muscle. Values are presented as mean ± SEM.

We subsequently focused on the potential factors related to the activation of insulin signaling and glycogen synthesis. At 120 min after ingestion, Akt phosphorylation and glycogen synthase (GS) phosphorylation status in the liver were not significantly different among the three groups (Figure 5A–C). Similarly, Akt and GS phosphorylation status were not significantly different in the plantaris muscle (Figure 5D–F) or soleus muscle (Figure 5G–I). 

#### 3.2.3. Energy Expenditure in the Recovery Period

**Figure 6:**
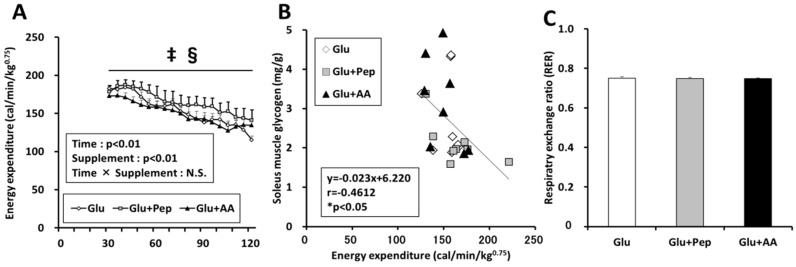
Energy expenditure during the recovery period. (**A**) Energy expenditure during the recovery period; (**B**) Correlation between energy expenditure and muscle glycogen content; (**C**) Respiratory exchange ratio. Values are presented as mean ± SEM. ^‡^ significantly different from the Glu group (*p* < 0.05). ^§^ significantly different from the Glu + AA group (*p* < 0.05).

Two-way ANOVA revealed that nutrient supplementation had a significant effect on energy expenditure during the recovery period, and the energy expenditure in the Glu + Pep group was significantly higher than that in the Glu and Glu + AA groups (Figure 6A, *p* < 0.05). Furthermore, a significant negative correlation was observed between energy expenditure and the soleus muscle glycogen content (Figure 6B, *p* < 0.05). However, the respiratory exchange ratio (RER) was similar among the three groups (Figure 6C).

#### 3.2.4. Energy Consumption State

**Figure 7:**
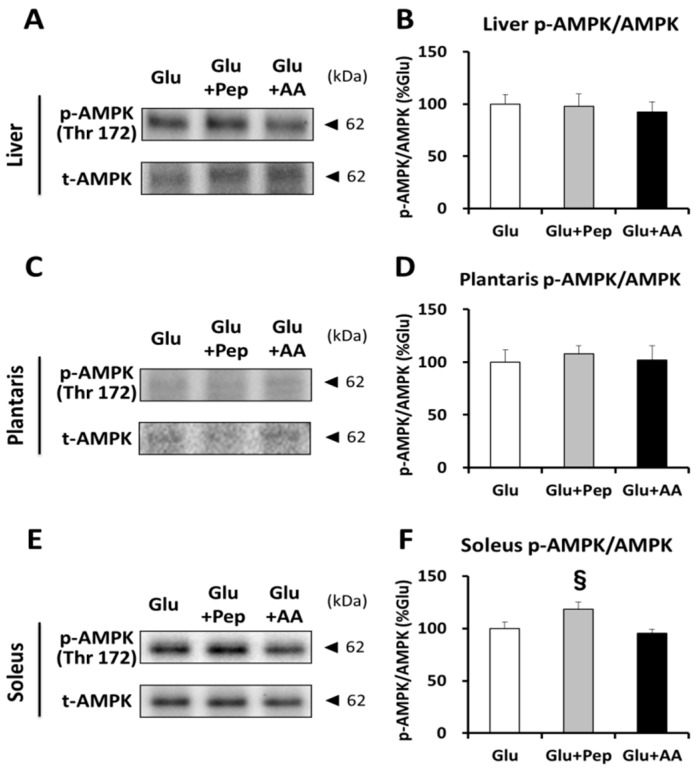
AMPK phosphorylation state. (**A**) Representative blots for p-AMPK and total-AMPK in the liver; (**B**) AMPK phosphorylation state in the liver; (**C**) Representative blots for p-AMPK and total-AMPK in the plantaris muscle; (**D**) AMPK phosphorylation state in the plantaris muscle; (**E**) Representative blots for p-AMPK and total-AMPK in the soleus muscle; (**F**) AMPK phosphorylation state in the soleus muscle. Values are presented as mean ± SEM. ^§^ significantly different from the Glu + AA group (*p* < 0.05).

Since ingestion of casein peptide with glucose enhanced energy expenditure, we further analyzed the AMPK phosphorylation state, as an index of the energy consumption state, in the liver, plantaris muscle, and soleus muscle. AMPK phosphorylation levels in the liver and plantaris muscle were similar among the three groups (Figure 7A–D). However, the soleus muscle AMPK phosphorylation level in the Glu + Pep group was significantly higher than that in the Glu + AA group (*p* < 0.05) and also tended (*p* = 0.078) to be higher than that in the Glu group (Figure 7E,F).

#### 3.2.5. Relation of Energy Expenditure and Muscle Glycogen Concentration with AMPK Phosphorylation in the Soleus Muscle

**Figure 8:**
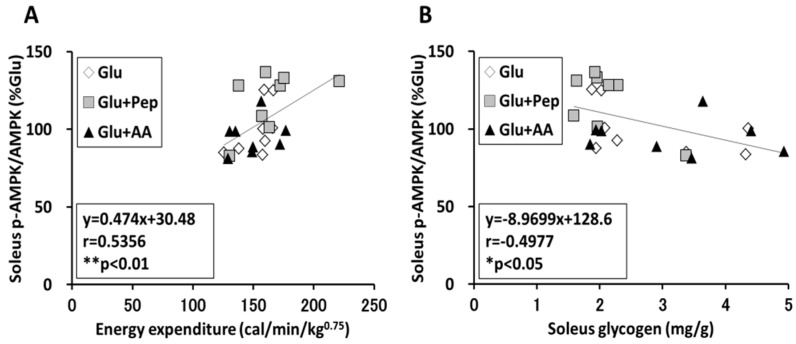
Correlation of energy expenditure, muscle glycogen content, and AMPK phosphorylation state in the soleus muscle. (**A**) Correlation between energy expenditure and AMPK phosphorylation state in the soleus muscle; (**B**) Correlation between muscle glycogen content and AMPK phosphorylation state in the soleus muscle. Values are presented as mean ± SEM.

A significant positive correlation was observed between energy expenditure and AMPK phosphorylation in the soleus muscle (Figure 8A, *p* < 0.01), while a significant negative correlation was observed between muscle glycogen content and AMPK phosphorylation (Figure 8B, *p* < 0.05).

## 4. Discussion

To the best of our knowledge, this is the first study to compare the effects of post-exercise ingestion of glucose plus casein peptide or that of its constituent amino acids on post-exercise muscle glycogen resynthesis. We showed that post-exercise ingestion of glucose with casein peptide (1) attenuated soleus muscle glycogen resynthesis; (2) did not affect insulin signaling and glycogen synthase phosphorylation; and (3) enhanced energy expenditure and AMPK phosphorylation, compared to that of glucose or glucose with amino acid.

The combination of carbohydrate and protein intake increases glycogen resynthesis, which has mainly been attributed to the enhancement of insulin secretion [6]. In this study, we confirmed that plasma insulin Cmax and AUC in the Glu + Pep (+22% and +38%) and Glu + AA (+38% and 15%) groups were increased compared with those of the Glu group, but the differences were not statistically significant. Similarly, there were no differences in Akt and glycogen synthase phosphorylation according to the supplementation treatment results. These results suggested that ingestion of casein peptide or its constituent amino acids does not markedly increase glycogen synthesis signaling. Previous studies reported that glucose with milk and whey isolate mixture [6], and whey hydrolysate [9] enhances insulin secretion. In addition, amino acid and wheat protein hydrolysate increased insulin levels [16] and muscle glycogen restoration, when compared with the same dose of carbohydrate intake [17]. Therefore, these results suggest that differences in amino acid composition or peptide sequences may affect insulin secretion, and that casein peptide co-ingestion with glucose does not have a strong synergistic effect on insulin secretion. Furthermore, one potential reason for the lack of an effect is the dosage applied. We selected a dose of 1.5 mg/g glucose for this study based on the results of a previous study [5], which confirmed the effects of 1.5 mg/g glucose ingestion and revealed that glycogen was appropriately resynthesized. In addition, Kerksick et al. [18] stated that both carbohydrate and protein (at a 3–4:1 ratio) could promote the recovery and replenishment of muscle glycogen based on a systematic review. Therefore, we selected the amount of glucose and peptide/amino acid (1.5 mg with 0.5 mg/g body weight) according to these previous findings. In contrast, through a review of the literature, Jentjens and Jeukendrup [19] indicated that maximal glycogen synthesis rates occur at a carbohydrate intake of ~1.2 g·kg^−1^·h^−1^. Indeed, 1.67 g/kg BW of sucrose with 0.5 g/kg BW of whey protein hydrolysate ingestion did not enhance glycogen resynthesis compared with the same dose of sucrose with water, although protein hydrolysate with sucrose enhanced insulin secretion [20]. Thus, it might have been difficult to obtain an additional effect on glycogen resynthesis via protein source ingestion when a sufficient amount of glucose was ingested. Moreover, fasting duration affects insulin secretion. The mice were starved for 16-h to decrease the glycogen content in this study, which is a longer fasting time than used in the previous study [9]. Fasting for 24 h reduced basal plasma insulin levels in both chow and high-fat-fed mice by three and five-fold, respectively, compared with fasting duration of 0 h [21]. Long-term fasting attenuates the insulin response in human study [22,23]. Thus, it is possible that the fasting time is related to insulin secretion and glycogen resynthesis.

Although glucose with protein source ingestion did not have a significant effect on insulin secretion and glycogen synthesis signaling compared with glucose alone, co-ingestion of glucose and casein peptide attenuated post-exercise glycogen recovery in the soleus muscle. The effect of casein on energy expenditure could explain this result. A part of the energy substrate ingested by meals is converted to heat, and diet-induced thermogenesis (DIT) varies depending on the food constituents. Raben et al. [24] reported that the average DIT was 17% higher after intake of a protein meal than after intake of carbohydrate and fat-rich meals. Casein hydrolysate intake was shown to enhance lipid utilization compared with intact casein, although the energy expenditure did not change [25], and we previously showed that pre-exercise casein peptide supplementation improved glucose tolerance [11]. Thus, casein peptide might affect energy metabolism. Indeed, we clarified that glucose along with casein peptide ingestion enhanced energy expenditure in recovery period and a significant negative correlation was observed between energy expenditure and the soleus muscle glycogen content. These results suggested that glycogen recovery was related to energy expenditure. Increasing energy expenditure activates AMPK. We previously reported that pre-exercise casein peptide ingestion enhanced AMPK phosphorylation and mitochondrial enzyme activity [26]. Similarly, we measured AMPK phosphorylation as an index of energy depletion and confirmed that glucose along with casein peptide ingestion enhanced AMPK phosphorylation in soleus muscle, but not in the liver and plantaris muscle. Furthermore, a previous study showed that treatment with 5-aminoimidazole-4-carboxamide riboside (AICAR), an AMPK activator, enhanced 3-*O*-methylglucose (3-MG) uptake, but suppressed glycogen synthase activity [27]. Therefore, casein peptide ingestion appears to have increased energy expenditure in the soleus muscle during the recovery period, which was partly related to the decline in muscle glycogen resynthesis.

We showed that co-ingestion of glucose and casein peptide attenuates glycogen recovery in soleus, but not plantaris muscle in this study. These results suggest that muscle glycogen recovery depends on the type of muscle. In addition, differences in exercise intensity and pattern might affect glycogen recovery; however, we only examined glycogen recovery using acute low-intensity endurance exercise in this study. High intensity exercise was reported to decrease the muscle glycogen content [28], and glycogen recovery after high-intensity exercise was similar to that detected after low-intensity exercise [29]. Enhancement of exercise intensity increases glucose utilization and decreases glycogen storage [2,3], also affects muscle AMPK signaling [30] and blood flow to each muscle [31]. In addition, strength training can enhance glucose uptake [32], and muscle glycogen supercompensation in endurance exercise-trained rats was reported to be higher than that in untrained rats [33]. Therefore, it is necessary to study the effect of supplementation on various exercise intensities and forms in the future.

There are several limitations of this study that should be noted. First, we could not measure the glucose uptake and energy expenditure for each tissue. Therefore, it is not clear whether the delayed glycogen recovery following co-ingestion of glucose and casein peptide was due to inhibition of glucose uptake or to excess consumption of energy for each tissue. Second, we could not clarify why ingestion of glucose with casein peptide enhanced the total energy expenditure. Previous studies suggested that ingested ^14^C-labeled methionine-lysine-proline tri-peptide could be detected in the blood [34] and reached many organs and tissues, including the liver and brain tissue [35]. In addition, a tryptophan-histidine di-peptide was found to enhance glucose uptake [36] and a hydroxyprolyl-glycine di-peptide induced myotube hypertrophy [37] in vitro. Therefore, it is possible that a casein-derived peptide would affect energy expenditure and glycogen resynthesis by reaching related tissues. Conversely, it is expected that bioactive peptide concentrations in the blood are extremely low. Therefore, it may act not only directly on the muscles, but also indirectly through the intestines. However, the precise mechanism and detection of peptides remain unclear, which should be explored in future studies.

## 5. Conclusions

Co-ingestion of glucose and casein peptide attenuated post-exercise glycogen recovery compared with only glucose and glucose with amino acids. One of the potential reasons for this effect might be related to the enhancement of energy expenditure during the recovery period. Therefore, casein peptide may be preferable for efficiently consuming energy such as for achieving an anti-obesity effect rather than for enhancing glycogen recovery.

## Figures and Tables

**Table 1:** Amino acid composition of casein peptide.

**Essential Amino Acids (mg/g)**			
Lysine	68	Leucine	72
Threonine	44	Phenylalanine	31
Valine	55	Tyrosine	33
Methionine	24	Tryptophan	2
Cysteine	3	Histidine	22
Isoleucine	46		
**Non-Essential Amino Acids (mg/g)**			
Aspartic acid + Asparagine	75	Glycine	19
Serine	56	Alanine	30
Glutamic acid + Glutamine	250	Arginine	22
Proline	96		
